# Mechanisms of magnoliae cortex on treating sarcopenia explored by GEO gene sequencing data combined with network pharmacology and molecular docking

**DOI:** 10.1186/s12863-022-01029-x

**Published:** 2022-02-17

**Authors:** Xingqi Zhao, Feifei Yuan, Haoyang Wan, Hanjun Qin, Nan Jiang, Bin Yu

**Affiliations:** 1 Division of Orthopaedics and Traumatology, Department of Orthopaedics & Guangdong Provincial Key Laboratory of Bone and Cartilage Regenerative Medicine, Nanfang Hospital, Southern Medical University, Guangzhou, 510515 China; 2 Department of Pediatrics, The Third Affiliated Hospital of Guangzhou Medical University, Guangzhou, 510150 China

**Keywords:** Sarcopenia, Magnoliae Cortex, Network pharmacology, Compound-target relationship, Gene ontology, KEGG, Molecular docking

## Abstract

**Background:**

Administration of Magnoliae Cortex (MC) could induce remission of cisplatin-induced sarcopenia in mice, however, whether it is effective on sarcopenia patients and the underlying mechanisms remain unclear.

**Methods:**

Sarcopenia related differentially expressed genes were analysed based on three Gene Expression Omnibus (GEO) transcriptome profiling datasets, which was merged and de duplicated with disease databases to obtain sarcopenia related pathogenic genes. Gene ontology (GO) and Kyoto Encyclopedia of Genes and Genomes (KEGG) analysis were than performed to analyse the role of proteins encoded by sarcopenia related pathogenic genes and the signal regulatory pathways involved in. The main active components and target proteins of MC were obtained by searching traditional Chinese medicine network databases (TCMSP and BATMAN-TCM). MC and sarcopenia related pathogenic genes shared target proteins were identified by matching the two. A protein–protein interaction network was constructed subsequently, and the core proteins were filtered according to the topological structure. GO and KEGG analysis were performed again to analyse the key target proteins and pathways of MC in the treatment of sarcopenia, and build the herbs-components-targets network, as well as core targets-signal pathways network. Molecular docking technology was used to verify the main compounds-targets.

**Results:**

Sarcopenia related gene products primarily involve in aging and inflammation related signal pathways. Seven main active components (Anonaine, Eucalyptol, Neohesperidin, Obovatol, Honokiol, Magnolol, and beta-Eudesmol) and 26 target proteins of MC-sarcopenia, of which 4 were core proteins (AKT1, EGFR, INS, and PIK3CA), were identified. The therapeutic effect of MC on sarcopenia may associate with PI3K-Akt signaling pathway, EGFR tyrosine kinase inhibitor resistance, longevity regulating pathway, and other cellular and innate immune signaling pathways.

**Conclusion:**

MC contains potential anti-sarcopenia active compounds. These compounds play a role by regulating the proteins implicated in regulating aging and inflammation related signaling pathways, which are crucial in pathogenesis of sarcopenia. Our study provides new insights into the development of a natural therapy for the prevention and treatment of sarcopenia.

**Supplementary Information:**

The online version contains supplementary material available at 10.1186/s12863-022-01029-x.

## Background

Sarcopenia is a progressive skeletal muscle disorder, characterized by low muscle strength, low muscle quantity/quality, as well as low physical performance according to the level of disease progression [1]. With the progression of sarcopenia, the incidence of adverse outcomes increases gradually, such as fractures [2, 3], physical disability [4], and mortality [5]. However, at present, there are limited preventive and therapeutic interventions for this disease [6].Therefore, new therapies for sarcopenia are urgently needed to intervene or delay adverse health outcomes.

One of the reasons why sarcopenia lacks effective treatment measures is that the pathogenesis is not fully understood, thus lack of intervention targets. Considering loss of muscle strength and mass is also a fundamental feature of aging, results of preclinical and clinical studies comparing young and aged individuals suggested that chronic low-grade inflammation contribute to a loss of muscle plasticity during aging [7]. It has been shown that NF-kB signaling and inflammatory cytokines also take part in the creation and maintenance of sarcopenia status [8]. Therefore, we speculated interventions against aging and inflammation may benefit sarcopenia. A recent in vivo study demonstrated that Magnoliae Cortex (MC), an herbal medicine widely used in medical practice of traditional Chinese medicine (TCM), could alleviate cisplatin-induced sarcopenia [9]. This result reminds us that MC might be a new drug to intervene and delay adverse consequences of sarcopenia.

As the wealth of China and the world, TCM has attracted more and more attention in the prevention and treatment of a series of diseases for the advantages of definite curative effect, safety, and few side effects. Different from the single targeted therapy of Western Medicine, herbal medicine of TCM mainly carries out multi-target treatment because they contain a large number of active chemical components. MC is called Houpu in Chinese herb (scientific term: *Magnolia Officinalis Rehd Et Wils.*), belongs to dampness removing drugs in TCM theory [10]. Recent pharmacological analysis have pointed out that MC has the effects of anxiolytic-like [11], apoptosis-inducing and antitumor [12], antimicrobial against multi-drug resistant *Staphylococcus aureus* [13], as well as lipid metabolism regulation [14]. Although previous studies have shown that MC can alleviate cisplatin-induced sarcopenia through immune regulation and inhibiting the expression of inflammatory cytokines [9], the specific active components, cellular and molecular mechanisms remain unclear. There are few or no systematic researches on the biological basis of TCM herbal medicine for treating sarcopenia. How to develop new methods to detect the key components for treating sarcopenia and speculate the possible mechanism not only provides the benefit therapy strategy for the precise treatment of sarcopenia, but also provides methodological reference for the analysis of the possible mechanisms.

Systems biology [15] and network pharmacology [16, 17] have been successfully applied in the targets prediction and mechanisms research in treatment of diseases with TCM. For example, Yang et al. used network pharmacology to decipher the cellular and molecular mechanisms of 8 different TCM formulas in the treatment of cardiovascular diseases [18]; Wang et al. expounded the molecular mechanism of 3 different TCM formulas in treating rheumatoid arthritis based on network pharmacology-based approach [19], etc. In recent years, system or network pharmacology combined with multi-omics analysis have shown unique advantages in predicting and interpreting the pharmacological principle of TCM herbs and their mechanism of action in treating various diseases [20–23].

**Fig. 1:**
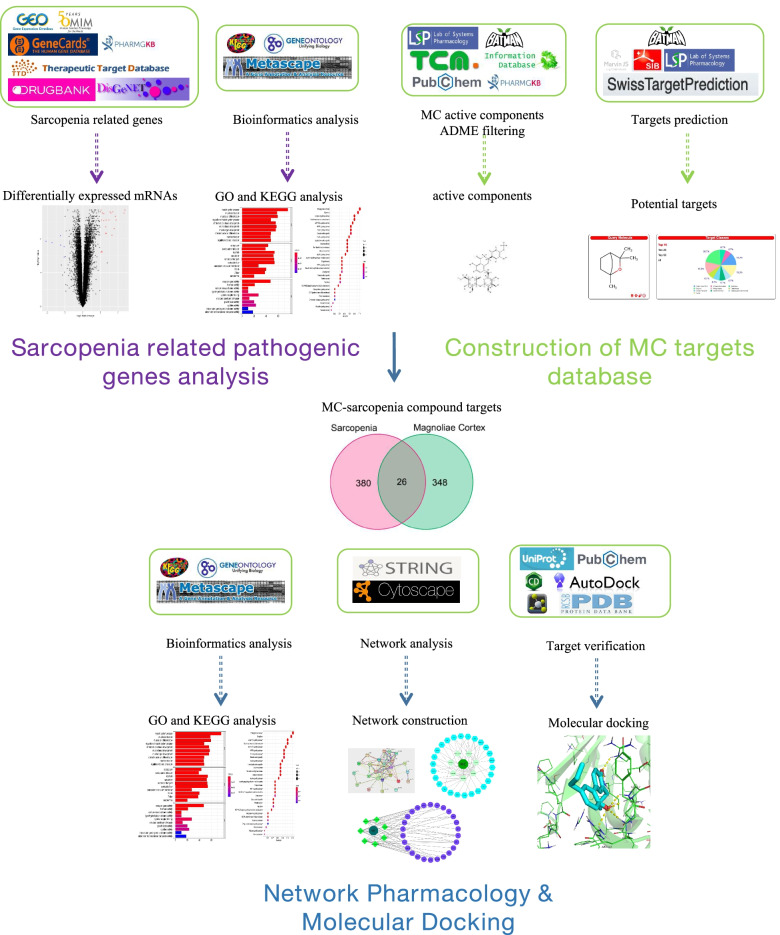
Research flow chart of the network pharmacological investigation on the use of MC in sarcopenia treatment

Under the premise of preclinical effectiveness in cisplatin-induced sarcopenia model, we wondered whether MC could also alleviate sarcopenia in clinical patients. In this study, we first looked for target genes/proteins that may interfere with the disease process through the sequencing data of sarcopenia muscle biopsies, and combined with the sarcopenia-related genes databases to obtain sarcopenia related pathogenic genes/proteins. Then, we used the network pharmacology method to predict the targets of MC, and matched them with sarcopenia related pathogenic genes to obtain MC-sarcopenia targets. Afterwards, the mechanism was systematically predicted according to protein functions and involved signal pathways. Finally, molecular docking technology was used to verify whether the active components of MC play a role in sarcopenia related pathogenic proteins. A research flow chart of the method was shown in Fig. 1.

## Materials and methods

### Construction of sarcopenia related pathogenic genes database

First, high-throughput sequencing data of mRNAs in the muscle biopsies of healthy and sarcopenia elderly people was obtained from the Gene Expression Omnibus (GEO) database (https://www.ncbi.nlm.nih.gov/geo/). We chose the following three series for analysis, including GSE111006, GSE111010, and GSE111016, as the individuals they included were the elderly with or without sarcopenia. Sva and Limma of R 3.6.3 were used to carry out data integration of multiple series and correct data batches effect. Genes with an adjusted *P* value < 0.05 and absolute value of log_2_(Fold Change) > 1 were considered as significantly differentially expressed and sarcopenia related pathogenic genes. In addition, sarcopenia related pathogenic genes were integrated with the disease-related genes database, including GeneCard database (https://www.genecards.org/), OMIM database (https://www.omim.org/), Pharmgkb (https://www.pharmgkb.org/), TTD database (http://db.idrblab.net/ttd/) [24], DrugBank database (https://go.drugbank.com/) [25], and DisGeNET database (https://www.disgenet.org) [26], using “sarcopenia” as keyword. Subsequently, the duplicated genes were removed, and the sarcopenia related pathogenic genes database was established.

### Gene ontology (GO) and Kyoto Encyclopedia of Genes and Genomes (KEGG) analysis

After obtaining the sarcopenia related pathogenic genes, we used ClusterProfiler [27] of R 3.6.3 to conduct the GO and KEGG analysis [28]. The related software packages can be obtained from https://www.bioconductor.org/. The GO enrichment mainly analyses the biological process (BP), cellular composition (CC), and molecular function (MF) of the genes, and the KEEG enrichment mainly analyses the potential biological pathways involved in these interested genes.

### Construction of MC main active components database

The TCM system pharmacology database and analysis platform (TCMSP, https://tcmspw.com/tcmsp.php) [29], the bioinformatics analysis tool for the molecular mechanism of TCM (BATMAN-TCM, http://bionet.ncpsb.org.cn/batman-tcm/) [30], and the TCM information database (http://bidd.group/TCMID/) [31] were used to identify the active components of MC. The main active components were then filtered according to the optimal toxicokinetic ADME rules [oral bioavailability (OB) ≥ 30%, drug-like (DL) ≥ 0.18] [32]. If the component did not meet the filtering criteria, they were included if they were reported as effective against sarcopenia in relevant literatures [9, 33]. The molecular structure of components was obtained from the PubChem (https://pubchem.ncbi.nlm.nih.gov/) or Pharmgkb (https://www.pharmgkb.org/).

### Construction of active components potential targets database

The components of herbal medicine perform related biological functions through relevant targets. In addition to obtaining the targets of the main active components of MC directly from the TCMSP and BATMAN-TCM, the Swiss Target Prediction (http://swisstargetprediction.ch/) [34] were also used to predict possible targets of MC.

### Construction of the Protein–Protein interaction (PPI) network

Based on the above analyses, the targets of main active components were matched with the disease-related pathogenic gene products of sarcopenia to obtain the compound targets of MC-sarcopenia. The Venn map was drawn by venn of R 3.6.3 and the PPI network of the targets was obtained by using the String online tool (https://string-db.org/) [35]. Then, the GO and KEGG analysis were conducted again to obtain the BP, CC, MF, and potential biological pathways of the compound targets.

### Construction of an “Herbs-Components-Targets” network of MC

Based on the PPI network obtained above, the “Herbs-Components-Targets” network (H-C-T network) of MC was constructed using Cytoscape3.8.2 (https://www.cytoscape.org/) [36]. According to the topological characteristics of the network, the follow three parameters were used to obtain the core composite targets of MC: degree centrality (DC) [37], closeness centrality (CC) [38], and betweenness centrality (BC) [39]. According to literature reports, the targets with higher than two-fold the median value of DC [40], BC and CC were considered as more accurate core targets [41].

### Active components-targets docking

According to the screened core targets, the active components that may bind to the core targets were searched in reverse to obtain the key components of MC, which were then docked with core targets to verify the accuracy of the main components and prediction targets. The candidate key components crystal structure and the core targets protein structure were downloaded from the PubChem and RCSB protein database (https://www.pdb.org/), respectively. The target proteins preferentially select the structure with molecular binding smaller than 3 Å. Then the protein was dehydrated, hydrogenated and ligand separated using Pymol 2.5.1 software (https://pymol.org/2/). The processed biological macromolecular protein was then poured into AutoDockTools 1.5.6 to construct the docking grid box [42, 43]. Molecular docking was completed by using Autodock Vina 1.1.2 software [44], and the molecule with the lowest binding energy in the docking conformation was used to observe the binding effect by matching with the original components and intermolecular interactions.

## Results

### Sarcopenia related pathogenic genes

**Fig. 2:**
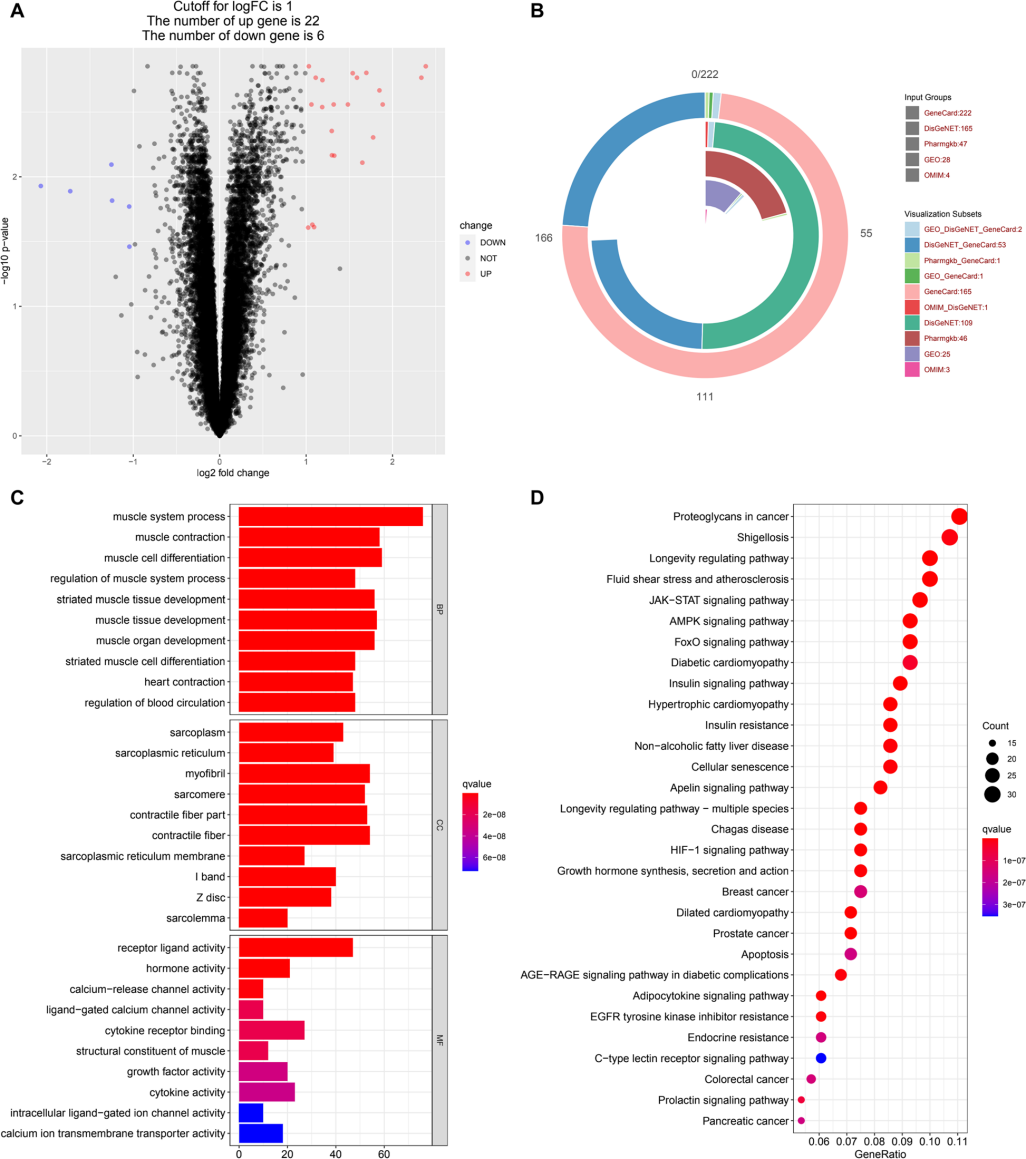
Sarcopenia related pathogenic genes. **A** Differential genes volcano map jointly analysed by three GEO series. The fold change of muscle biopsies mRNA in sarcopenia group compared with control group. **B** Integrated disease-related pathogenic genes in GeneCard, OMIM, Pharmgkb, DisGeNET, and GEO series. **C** The GO enrichment analysis of sarcopenia related pathogenic gene products. **D** The KEGG enrichment analysis of sarcopenia related pathogenic gene products

Joint analysis of three series in the GEO database (GSE111006, GSE111010, GSE111016) identified 28 differentially expressed genes related to sarcopenia in old people (Supplementary Table S1), which were used to build a volcano map (Fig. 2A). In addition, we integrated disease-related pathogenic genes in GeneCard, OMIM, Pharmgkb, and DisGeNET databases to eliminate duplicates, resulting in the identification of 406 sarcopenia-related pathogenic genes (Supplementary Table S2, Fig. 2B).

GO enrichment analysis was conducted for the identified 406 sarcopenia-related pathogenic genes on CC, MF and BP We selected the top 20 functional enrichment processes to draw a bar diagram (Fig. 2C). In terms of molecular function, also called the biochemical activity of gene products, sarcopenia-related pathogenic gene products mainly involve in the activity regulation of ligand, hormone, channel, receptor, cytokine, such as receptor ligand activity (GO:0,048,018), cytokine activity (GO:0,005,125) (Fig. 2C). Also, sarcopenia-related pathogenic gene products take part in the phosphatidylinositol 3-kinase activity (GO:0,035,004) and 1-phosphatidylinositol-3-kinase activity (GO:0,016,303) (Supplementary Table S3). In the biological process, sarcopenia-related pathogenic gene products mainly involves the system process and cell differentiation of muscle, such as muscle system process (GO:0,003,012), muscle cell differentiation (GO:0,042,692), regulation of muscle system process (GO:0,090,257) (Fig. 2C). Also, sarcopenia-related pathogenic gene products participate in the regulation of inflammatory response (GO:0,050,727), inflammatory cell apoptotic process (GO:0,006,925), regulation of protein kinase B signaling (GO:0,051,896) (Supplementary Table S3).

In addition, we identified the primary signaling pathways involved in sarcopenia by KEGG enrichment analysis, and filtered the top 20 pathways related to sarcopenia (adjusted *P* < 0.05), including longevity regulating pathway (hsa04211), EGFR tyrosine kinase inhibitor resistance (hsa01521), AMPK signaling pathway (hsa04152), Insulin resistance (hsa04931), FoxO signaling pathway (hsa04068), PI3K-Akt signaling pathway (hsa04151), endocrine resistance (hsa01522) among others (Fig. 2D, Supplementary Table S4). We listed sarcopenia related pathogenic gene products in several main signaling pathways, and found most of them play important role in related pathways (Supplementary Figures S1-S3).

### Active components and target prediction of MC

**Fig. 3:**
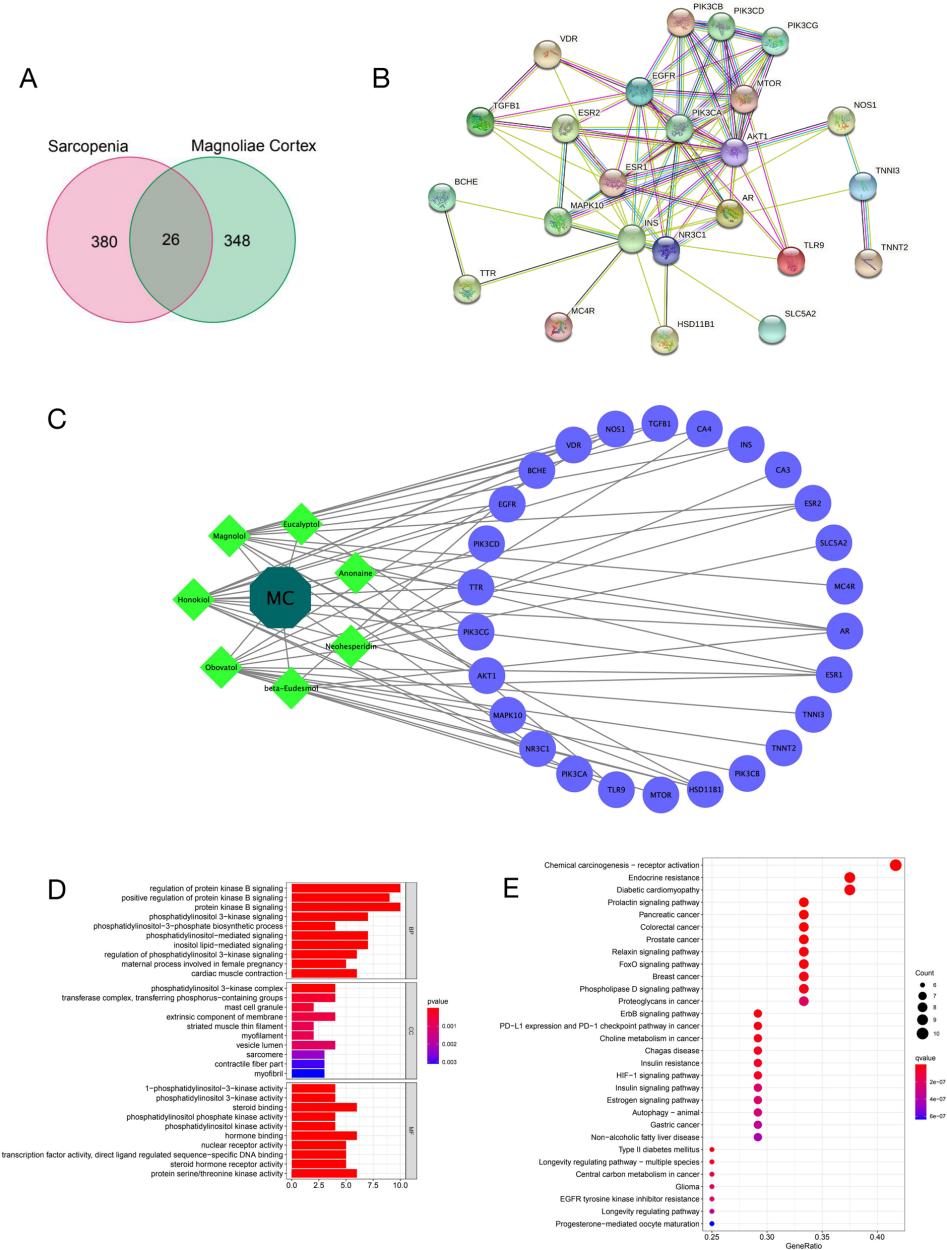
H-C-T network of MC-sarcopenia composite targets. **A** Venn diagram of the targets in MC and sarcopenia-related pathogenic genes. **B** PPI network of MC-sarcopenia composite targets. **C** H-C-T network. **D** The GO analysis of target proteins involved in sarcopenia treatment by MC. **E** The KEGG analysis of target protein signal pathway involved in sarcopenia treatment by MC

A total of 184 active components were obtained from TSMSP, BATMAN-TCM, and TCMID, and four main active components were selected according to the filtering criteria of ADME (OB ≥ 30% and DL ≥ 0.18). However, Honokiol and Magnolol were verified as two major active components in MC using high pressure liquid chromatography (HPLC, approximately 0.8% and 2.1% in MC respectively), and related literature confirmed that they showed protective effects in an experimental sarcopenia animal model [9]. In addition, beta-Eudesmol is one of the most studied and major bioactive sesquiterpenes, showed therapeutic potential and pharmacological activities in a series of diseases [33]. Therefore, they were also included although they did not meet the ADME criteria. Finally, seven main active components were included (Table 1). Then, 374 MC target proteins were identified by integrating the data obtained from TCMSP, BATMAN-TCM, and Swiss Target Prediction (Probability > 0.05) (Supplementary Table S5). These target proteins of MC were matched with sarcopenia-related pathogenic gene products, resulting in the selection of 26 composite targets of MC and sarcopenia (Fig. 3A, Supplementary Table S6).

**Table 1:** Main components of MC

PubChem CID	Molecule Name	OB (%)	DL	Structure
160597	Anonaine	25.14	0.47	
2758	Eucalyptol	60.62	0.32	
442439	Neohesperidin	57.44	0.27	
100771	Obovatol	69.45	0.18	
72303	Honokiol	60.67	0.15	
72300	Magnolol	69.199	0.15	
91457	beta-Eudesmol	26.09	0.10

### H-C-T network of MC-sarcopenia composite targets

The MC-sarcopenia composite targets identified were input into STRING to remove the unconnected targets, and the PPI network was obtained (Fig. 3B). Then, the H-C-T network of MC was constructed using Cytoscape 3.8.2 (Fig. 3C), including 34 nodes and 55 edges.

GO enrichment analysis showed that the active components of MC involve in affecting the phosphatidylinositol 3-kinase activity (GO:0,035,004), 1-phosphatidylinositol-3-kinase activity (GO:0,016,303) of sarcopenia. Proteins affected by MC active components participate in the regulation of protein kinase B signaling (GO:0,051,896), response to steroid hormone (GO:0,048,545), inflammatory cell apoptotic process (GO:0,006,925), positive regulation of inflammatory response (GO:0,050,729), and regulation of inflammatory response (GO:0,050,727) as well (Fig. 3D, Supplementary Table S7).

KEGG enrichment analysis showed that proteins affected by MC active components mainly participate in endocrine resistance (hsa01522), FoxO signaling pathway (hsa04068), PI3K-Akt signaling pathway (hsa04151), EGFR tyrosine kinase inhibitor resistance (hsa01521), and longevity regulating pathway(hsa04211), etc. (Fig. 3E, Supplementary Table S8). These signaling pathways also play important role in the pathogenesis of sarcopenia, further indicate that MC can be used in the treatment of sarcopenia.

### Molecular docking analysis

**Fig. 4:**
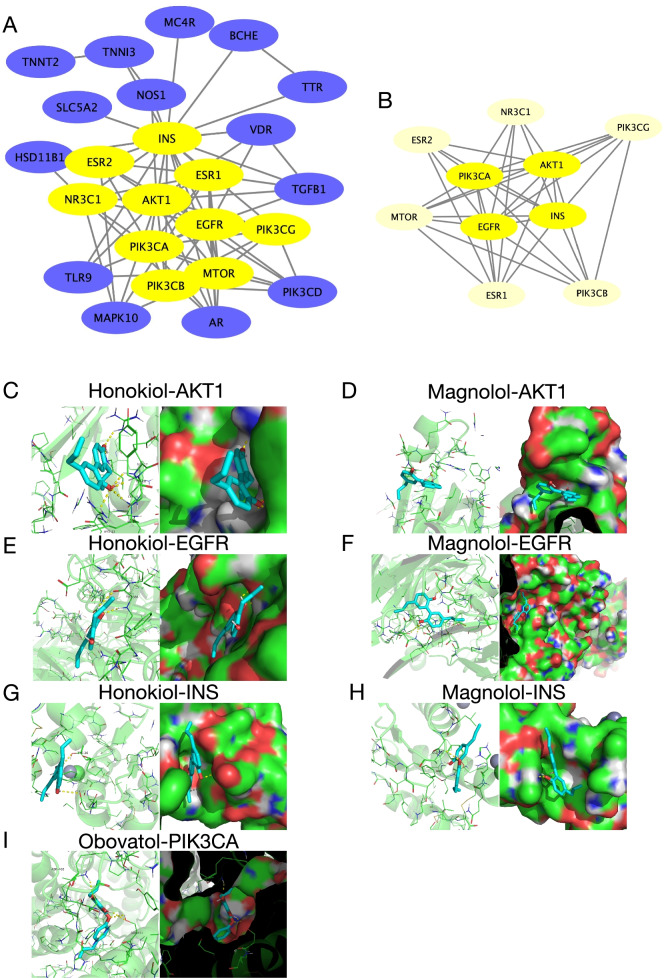
The protein–ligand of the docking simulation. **A-B** The process of topological filtering for core proteins of sarcopenia. **C** Honokiol-AKT1. **D** Magnolol-AKT1. **E** Honokiol-EGFR. **F** Magnolol-EGFR. **G** Honokiol-INS. **H** Magnolol-INS. **I** Obovatol-PIK3CA

In order to verify the above analysis results, we conducted molecular docking for the active components of MC and sarcopenia-related pathogenic proteins. Firstly, we filtered the core proteins of MC-sarcopenia composite targets according to the characteristics of the network topology, using NetworkAnalyzer plug-in unit of Cytoscape (Fig. 4A). After twice filtering, we obtained four core proteins, including AKT1, EGFR, INS, and PIK3CA (Fig. 4B, Supplementary Table S9). Consistent with the sarcopenia-related pathogenic proteins, these four core proteins are mainly involved in PI3K-Akt signaling pathway (hsa04151) and longevity regulating pathway (hsa04213) (Supplementary Figures S4-S6). In retrospect, we matched the targets of active components in the MC corresponding to these four core proteins. Then, these active components of MC were selected for molecular docking verification, including Honokiol, Magnolol, and Obovatol (Table 2).

**Table 2:** MC active components that may have effects on selected core proteins

Molecule Name	Gene symbol	Protein name	PDB Entry	Protein crystal structure
Honokiol, Magnolol	AKT1	RAC-alpha serine/ threonine-protein kinase	1UNQ	
Honokiol, Magnolol	EGFR	Epidermal growth factor receptor	3P0Y	
Honokiol, Magnolol	INS	Insulin	1EV3	
Obovatol	PIK3CA	Phosphatidylinositol 4,5-bisphosphate 3-kinase catalytic subunit alpha isoform	5FI4	

The affinity energy of best mode for Honokiol-AKT1 and Magnolol-AKT1 were − 6.2 kcal/mol and − 6.7 kcal/mol, respectively (Supplementary Table S10, S11). Hydrogen bonding plays a key role in molecular recognition and biology. The result of Honokiol-AKT1 molecular docking showed that there were six hydrogen bondings formed by lysine residues (LYS-14), glutamicacid residues (GLU-17), tyrosine residues (TYR-18), isoleucine residues (ILE-19), arginine residues (ARG-23), arginine residues (ARG-86) in AKT1 protein with Honokiol crystal structure (Fig. 4C). The molecular docking of Magnolol-AKT1 showed one hydrogen bonding formation between tyrosine residues (TYR-38) in AKT1 protein and Magnolol crystal structure (Fig. 4D).

In the process of docking with EGFR, the affinity energy of best mode for Honokiol-EGFR and Magnolol-EGFR were − 7.0 kcal/mol and − 7.4 kcal/mol, respectively (Supplementary Table S12, S13). The molecular docking of Honokiol-EGFR showed two hydrogen bondings formation between tryptophan residues (TRP-386) in EGFR protein and Honokiol crystal structure (Fig. 4E). The result of Magnolol-EGFR molecular docking showed that there were three hydrogen bondings formed by alanine residues (ALA-40), glycine residues (GLY-42), lysine residues (LYS-42) in EGFR protein with Magnolol crystal structure (Fig. 4F).

The affinity energy of best mode for Honokiol-INS and Magnolol-INS were − 6.3 kcal/mol and − 6.0 kcal/mol, respectively (Supplementary Table S14, S15). The result of Honokiol-INS molecular docking showed that there were two hydrogen bondings formed by glutamicacid residues (GLU-21), tyrosine residues (TYR-26) in INS protein with Honokiol crystal structure (Fig. 4G). The molecular docking of Magnolol-INS showed one hydrogen bonding formation between glutamicacid residues (GLU-21) in INS protein and Magnolol crystal structure (Fig. 4H).

In the process of docking with PIK3CA, the affinity energy of best mode for Obovatol-PIK3CA was − 7.4 kcal/mol (Supplementary Table S15). The molecular docking of Obovatol-PIK3CA showed three hydrogen bondings formation between asparagine residues (ASN-465), serine residues (SER-474) in PIK3CA protein and Obovatol crystal structure (Fig. 4I).

## Discussion

Optimal intervention for people with sarcopenia is essential because the condition has not only high personal, but also social and economic burdens when untreated [45]. Presence of sarcopenia increases risks for hospitalisation, as well as the cost of care during hospitalisation [46–49]. Recent study showed MC could alleviate muscle wasting in a cisplatin-induced sarcopenia mouse model [9]. However, its effects on sarcopenia patients, as well as potential mechanism, have not been investigated yet. In the current study, results of bioinformatics analysis and network pharmacology analysis showed that main active components of MC target the core proteins of PI3K-Akt signaling pathway, EGFR tyrosine kinase inhibitor resistance, longevity regulating pathway, which may play a certain therapeutic role in sarcopenia. Furthermore, the results of molecular docking showed that there exists direct hydrogen bondings between the active components (Honokiol, Magnolol, and Obovatol) of MC and the core proteins of sarcopenia (AKT1, EGFR, INS, and PIK3CA), which verifies our analysis and prediction from another angle. We provided a series of pharmaceutical active ingredients that may be used to treat sarcopenia and speculated their possible mechanisms.

The limited mechanistic understanding of sarcopenia pathophysiology is one of the major reasons why sarcopenia lacks effective treatment measures, thus lack of molecular targets. Previous investigations comparing skeletal muscle in the elderly with that in the young adults have identified mechanisms that drive muscle aging without distinction for the mechanisms that specifically lead to pathological decline and physical disability [50–52]. With the recognition that sarcopenia is a specific pathological disorder no matter in the elderly or the young adults [1], we included three muscle biopsies sequencing data (GSE111006, GSE111010, GSE111016) from GEO database to analyse the difference expressed mRNAs between sarcopenia and non-sarcopenia in elderly patients. After integrating the above sequencing data results with the confirmed candidate genes of sarcopenia in GeneCard, OMIM, Pharmgkb, and DisGeNET databases, we obtained sarcopenia related pathogenic genes (Supplementary Table S2). The sarcopenia related pathogenic genes of our bioinformatics analysis result contains many differentially expressed genes detected by RT-PCR method in the study of Patel et al. [53]. According to the results of GO and KEGG enrichment analyses, sarcopenia related pathogenic gene products primarily involve in aging and inflammation related signal pathways, such as longevity regulating pathway (hsa04211), cellular senescence (hsa04218), TNF signaling pathway (hsa04668), IL-17 signaling pathway (hsa04657), EGFR tyrosine kinase inhibitor resistance (hsa01521), PI3K-Akt signaling pathway (hsa04151), and endocrine resistance (hsa01522) et al.. These sarcopenia related signaling pathways were similar to those explored in recent years. Wilson et al. considered that age-related decline in immune cell function, increased inflammation and the dysregulation of the PI3K-Akt pathway in neutrophils could contribute pathogenically to sarcopenia [54]. Furthermore, inflammaging, characterized by increased levels of proinflammatory cytokines and a reduced level of anti-inflammatory cytokines, contributes to the creation and maintenance of sarcopenia states [55]. In addition, a recent preclinical study has shown that intervention against inflammatory response could effectively alleviate the symptoms of sarcopenia [9]. The drug used in this study was traditional herbal medicine MC of TCM. We thus wonder whether this herbal medicine can be used in the treatment of sarcopenia patients.

Through the method of network pharmacology, we obtained the active components and potential intervention targets of MC. By matching the drug targets of MC with sarcopenia related pathogenic proteins, we obtained the related proteins of MC involved in sarcopenia intervention, namely MC-sarcopenia targets. After GO and KEGG enrichment analyses performed for these MC-sarcopenia targets, we found that proteins affected by MC active components participate in a large number of key sarcopenia related pathogenic signaling pathways, such as endocrine resistance (hsa01522), PI3K-Akt signaling pathway (hsa04151), EGFR tyrosine kinase inhibitor resistance (hsa01521), longevity regulating pathway(hsa04211), etc. (Fig. 3E, Supplementary Table S7). These results suggest that MC is likely to be a promising therapeutic drug for sarcopenia. Then, MC-sarcopenia targets were filtered to obtain four core proteins, namely PIK3CA, AKT1, EGFR, and INS. As PIK3CA and AKT1 are the core components of PI3K-Akt signaling pathway, we speculate that the mechanism of MC participating in sarcopenia treatment may be through the regulation of PI3K-Akt signaling pathway, which also play crucial role in inflammaging [54]. Finally, we used molecular docking technology to verify whether the active components in MC can interact with sarcopenia related core proteins. As is shown in Fig. 4, there exists at least one hydrogen bonding between residues of sarcopenia related core proteins and MC active components. Surprisingly, there were six hydrogen bondings formed by residues in AKT1 crystal structure with Honokiol (Fig. 4C). Therefore, we speculate the therapeutic effect of MC on sarcopenia may play a role in the physiological function of AKT1 through Honokiol. However, this needs further research, as well as verification.

A major limitation of the current study is that our results are based on existing databases. Thus, our findings need further validation in cell, animal experiments, and clinical trials, ultimately. First, we need to conduct cellular (in vitro) and animal (in vivo) experiments to verify whether MC has the effects of preventing and treating sarcopenia. Subsequently, it can be grouped according to different MC active components to filter the active components with better anti-sarcopenia effect, so as to clarify the exact active monomer component or component combinations of anti-sarcopenia in MC. In future research, we should also clarify the following issues: cellular and molecular mechanisms of MC active components in the treatment of sarcopenia, optimal dose of MC active components for inducing remission with low toxicity, and whether MC is suitable for long-term maintenance treatment of sarcopenia. We hope that we could finally find a monomer component or combination with exact anti-sarcopenia effect and clarify its potential action mechanism, which can be applied to clinic practice and alleviate the current situation of lack of anti-sarcopenia drugs.

## Conclusions

MC might be a promising therapeutic drug for sarcopenia. MC contains potential anti-sarcopenia active compounds. These compounds play a role by regulating the proteins implicated in regulating aging and inflammation related signaling pathways, which are crucial in pathogenesis of sarcopenia. The molecular mechanism underlying the effect of MC on inducing sarcopenia remission was predicted using a network pharmacology method, thereby providing a theoretical basis for further study of the effective components and mechanism of MC in the treatment of sarcopenia.

## Supplementary Information

**Additional file 1: Table S1.** Differentially expressed genes related to sarcopenia in old people of GEO series.**Table S2.** Integrated data of sarcopenia related pathogenic genes. **Table S3.** Result of GO enrichment analysis for sarcopenia related pathogenic gene products. **Table S4.** Result of KEGG enrichment analysis for sarcopenia related pathogenic gene products. **Table S5.** Result of targets prediction of MC. **Table S6.** Composite targets of MC and sarcopenia. **Table S7.** Result of GO enrichment analysis for composite targets of MC and sarcopenia. **Table S8.** Result of KEGG enrichment analysis for composite targets of MC and sarcopenia. **Table S9.** Core proteins of MC-sarcopenia composite targets. **Table S10.** The affinity energy of Honokiol-AKT1. **Table S11.** The affinity energy of Magnolol-AKT1. **Table S12.** The affinity energy of Honokiol-EGFR. **Table S13.** The affinity energy of Magnolol-EGFR. **Table S14.** The affinity energy of Honokiol-INS. **Table S15.** The affinity energy of Magnolol-INS. **Table S16.** The affinity energy of Obovatol-PIK3CA. **Figure S1.** Sarcopenia related pathogenic gene products involve in EGFR tyrosine kinase inhibitor resistance (hsa01521). **Figure S2.** Sarcopenia related pathogenic gene products involve in endocrine resistance (hsa01522). **Figure S3.** Sarcopenia related pathogenic gene products involve in longevity regulating pathway (hsa04211). **Figure S4.** The GO and KEGG analysis of core sarcopenia-related pathogenic proteins. **Figure S5.** Core sarcopenia related pathogenic gene products involve in PI3K-Akt signaling pathway (hsa04151). **Figure S6.** Core sarcopenia related pathogenic gene products involve in longevity regulating pathway (hsa04213).

## Data Availability

All the data can be obtained from the open-source website we provide, and the conclusion can be drawn through the analysis of the relevant software. The datasets generated and/or analysed during the current study are available in the Gene Expression Omnibus (GEO) database repository (GSE111006, https://www.ncbi.nlm.nih.gov/geo/query/acc.cgi?acc=GSE111006; GSE111010, https://www.ncbi.nlm.nih.gov/geo/query/acc.cgi?acc=GSE111010; GSE111016, https://www.ncbi.nlm.nih.gov/geo/query/acc.cgi?acc=GSE111016), which were used to analyse the differentially expressed genes in muscle tissue.

## Abbreviations

MC: Magnoliae Cortex
GEO: Gene Expression Omnibus
OMIM: Online Mendelian Inheritance in Man
TTD: Therapeutic Target Database
GO: Gene Ontology
KEGG: Kyoto Encyclopedia of Genes and Genomes
BP: Biological process
CC: Cellular composition
MF: Molecular function
TCM: Traditional Chinese Medicine
TCMSP: Traditional Chinese Medicine Systems Pharmacology Database
TCMID: Traditional Chinese Medicine Information Database
BATMAN-TCM: Bioinformatics analysis tool for the molecular mechanism of traditional Chinese medicine
ADME: Absorption, distribution, metabolism, and excretion
OB: Oral bio-availability
DL: Drug-likeness
PPI: Protein–protein interaction
H-C-T network: “Herbs-Components-Targets” network
DC: Degree Centrality
CC: Closeness Centrality
BC: Betweenness Centrality
