# Supplementary figures and images for: Mechanisms of magnoliae cortex on treating sarcopenia explored by GEO gene sequencing data combined with network pharmacology and molecular docking
Source: BMC Genom Data. 2022 Feb 17;23:15. doi: 10.1186/s12863-022-01029-x (PMC8851866; doi:10.1186/s12863-022-01029-x)

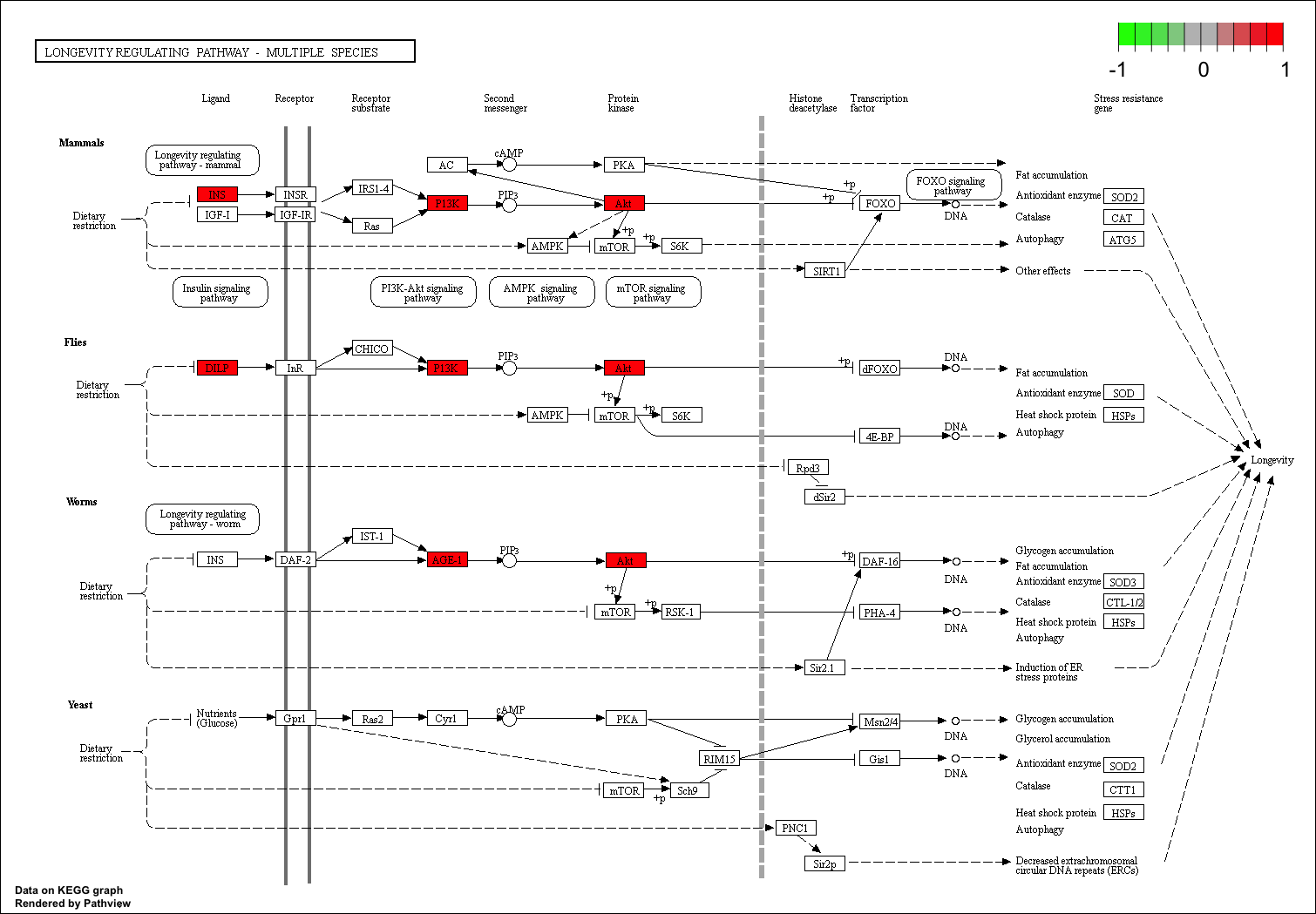

Supplement: Supplementary file 1 — Additional file 1: Table S1. Differentially expressed genes related to sarcopenia in old people of GEO series.Table S2. Integrated data of sarcopenia related pathogenic genes. Table S3. Result of GO enrichment analysis for sarcopenia related pathogenic gene products. Table S4. Result of KEGG enrichment analysis for sarcopenia related pathogenic gene products. Table S5. Result of targets prediction of MC. Table S6. Composite targets of MC and sarcopenia. Table S7. Result of GO enrichment analysis for composite targets of MC and sarcopenia. Table S8. Result of KEGG enrichment analysis for composite targets of MC and sarcopenia. Table S9. Core proteins of MC-sarcopenia composite targets. Table S10. The affinity energy of Honokiol-AKT1. Table S11. The affinity energy of Magnolol-AKT1. Table S12. The affinity energy of Honokiol-EGFR. Table S13. The affinity energy of Magnolol-EGFR. Table S14. The affinity energy of Honokiol-INS. Table S15. The affinity energy of Magnolol-INS. Table S16. The affinity energy of Obovatol-PIK3CA. Figure S1. Sarcopenia related pathogenic gene products involve in EGFR tyrosine kinase inhibitor resistance (hsa01521). Figure S2. Sarcopenia related pathogenic gene products involve in endocrine resistance (hsa01522). Figure S3. Sarcopenia related pathogenic gene products involve in longevity regulating pathway (hsa04211). Figure S4. The GO and KEGG analysis of core sarcopenia-related pathogenic proteins. Figure S5. Core sarcopenia related pathogenic gene products involve in PI3K-Akt signaling pathway (hsa04151). Figure S6. Core sarcopenia related pathogenic gene products involve in longevity regulating pathway (hsa04213). [file 12863_2022_1029_MOESM1_ESM.zip › Supplementary Figure S6.png]

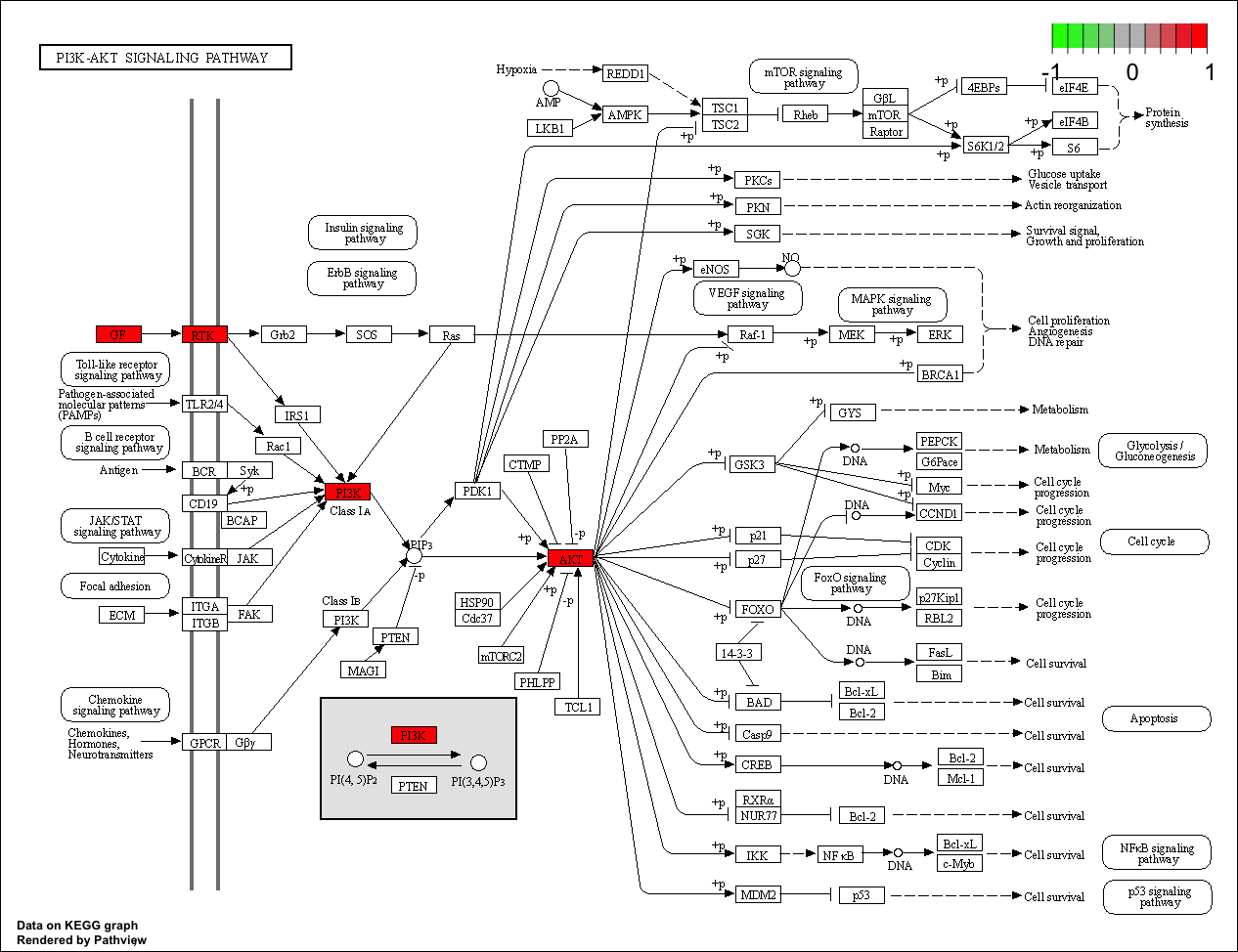

Supplement: Supplementary file 1 — Additional file 1: Table S1. Differentially expressed genes related to sarcopenia in old people of GEO series.Table S2. Integrated data of sarcopenia related pathogenic genes. Table S3. Result of GO enrichment analysis for sarcopenia related pathogenic gene products. Table S4. Result of KEGG enrichment analysis for sarcopenia related pathogenic gene products. Table S5. Result of targets prediction of MC. Table S6. Composite targets of MC and sarcopenia. Table S7. Result of GO enrichment analysis for composite targets of MC and sarcopenia. Table S8. Result of KEGG enrichment analysis for composite targets of MC and sarcopenia. Table S9. Core proteins of MC-sarcopenia composite targets. Table S10. The affinity energy of Honokiol-AKT1. Table S11. The affinity energy of Magnolol-AKT1. Table S12. The affinity energy of Honokiol-EGFR. Table S13. The affinity energy of Magnolol-EGFR. Table S14. The affinity energy of Honokiol-INS. Table S15. The affinity energy of Magnolol-INS. Table S16. The affinity energy of Obovatol-PIK3CA. Figure S1. Sarcopenia related pathogenic gene products involve in EGFR tyrosine kinase inhibitor resistance (hsa01521). Figure S2. Sarcopenia related pathogenic gene products involve in endocrine resistance (hsa01522). Figure S3. Sarcopenia related pathogenic gene products involve in longevity regulating pathway (hsa04211). Figure S4. The GO and KEGG analysis of core sarcopenia-related pathogenic proteins. Figure S5. Core sarcopenia related pathogenic gene products involve in PI3K-Akt signaling pathway (hsa04151). Figure S6. Core sarcopenia related pathogenic gene products involve in longevity regulating pathway (hsa04213). [file 12863_2022_1029_MOESM1_ESM.zip › Supplementary Figure S5.png]

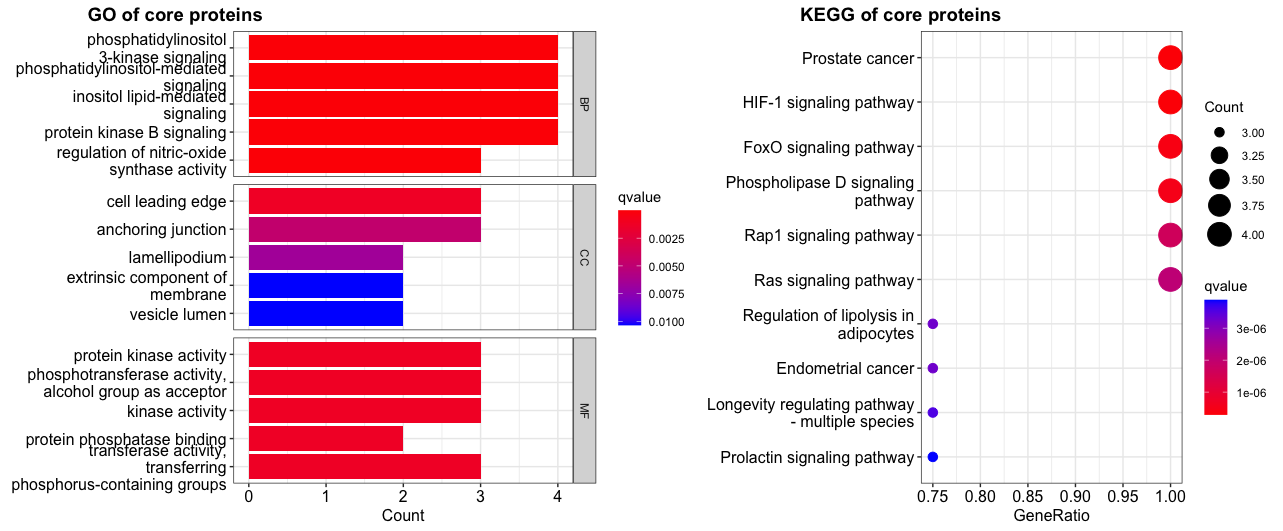

Supplement: Supplementary file 1 — Additional file 1: Table S1. Differentially expressed genes related to sarcopenia in old people of GEO series.Table S2. Integrated data of sarcopenia related pathogenic genes. Table S3. Result of GO enrichment analysis for sarcopenia related pathogenic gene products. Table S4. Result of KEGG enrichment analysis for sarcopenia related pathogenic gene products. Table S5. Result of targets prediction of MC. Table S6. Composite targets of MC and sarcopenia. Table S7. Result of GO enrichment analysis for composite targets of MC and sarcopenia. Table S8. Result of KEGG enrichment analysis for composite targets of MC and sarcopenia. Table S9. Core proteins of MC-sarcopenia composite targets. Table S10. The affinity energy of Honokiol-AKT1. Table S11. The affinity energy of Magnolol-AKT1. Table S12. The affinity energy of Honokiol-EGFR. Table S13. The affinity energy of Magnolol-EGFR. Table S14. The affinity energy of Honokiol-INS. Table S15. The affinity energy of Magnolol-INS. Table S16. The affinity energy of Obovatol-PIK3CA. Figure S1. Sarcopenia related pathogenic gene products involve in EGFR tyrosine kinase inhibitor resistance (hsa01521). Figure S2. Sarcopenia related pathogenic gene products involve in endocrine resistance (hsa01522). Figure S3. Sarcopenia related pathogenic gene products involve in longevity regulating pathway (hsa04211). Figure S4. The GO and KEGG analysis of core sarcopenia-related pathogenic proteins. Figure S5. Core sarcopenia related pathogenic gene products involve in PI3K-Akt signaling pathway (hsa04151). Figure S6. Core sarcopenia related pathogenic gene products involve in longevity regulating pathway (hsa04213). [file 12863_2022_1029_MOESM1_ESM.zip › Supplementary Figure S4.png]

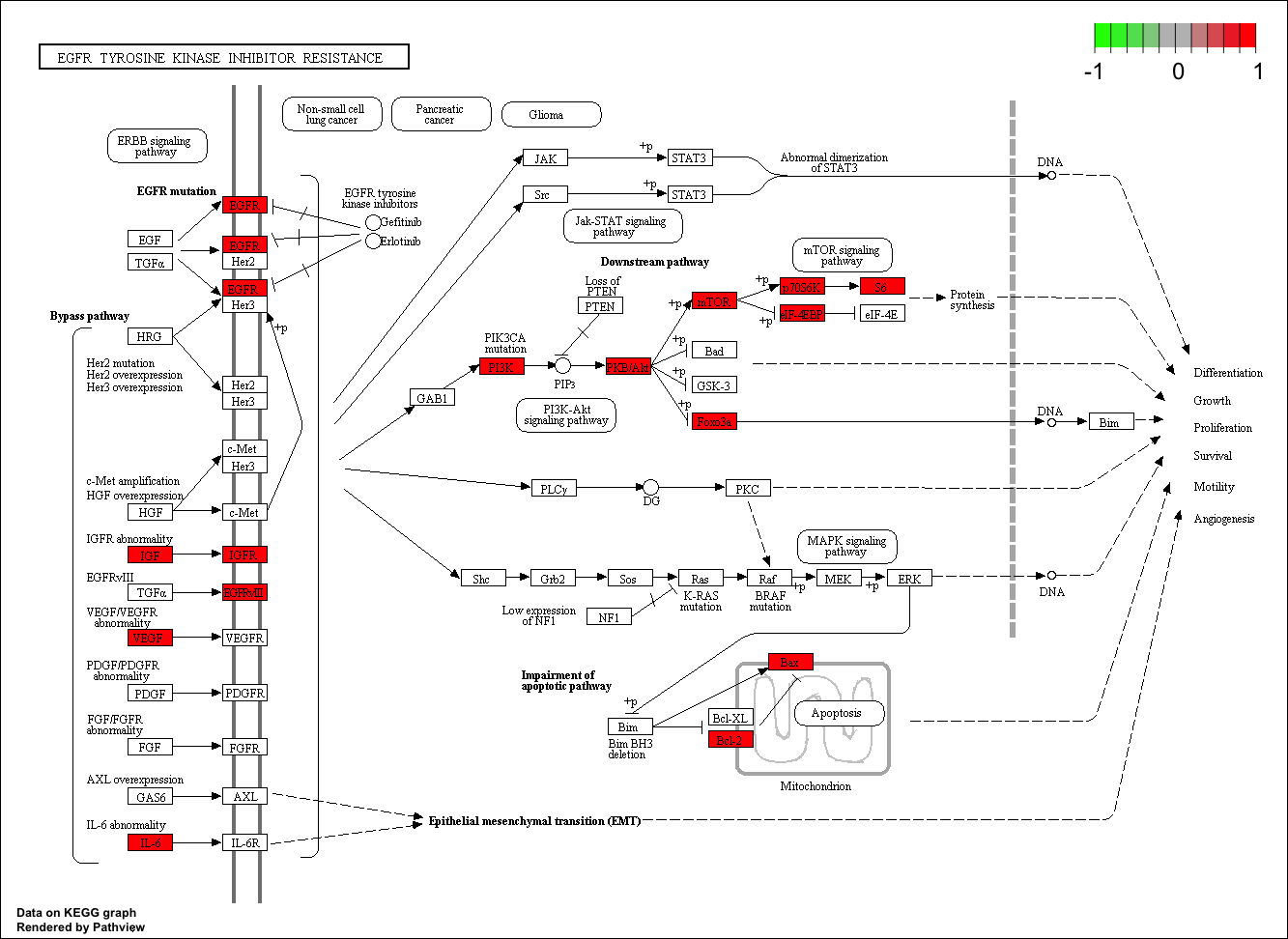

Supplement: Supplementary file 1 — Additional file 1: Table S1. Differentially expressed genes related to sarcopenia in old people of GEO series.Table S2. Integrated data of sarcopenia related pathogenic genes. Table S3. Result of GO enrichment analysis for sarcopenia related pathogenic gene products. Table S4. Result of KEGG enrichment analysis for sarcopenia related pathogenic gene products. Table S5. Result of targets prediction of MC. Table S6. Composite targets of MC and sarcopenia. Table S7. Result of GO enrichment analysis for composite targets of MC and sarcopenia. Table S8. Result of KEGG enrichment analysis for composite targets of MC and sarcopenia. Table S9. Core proteins of MC-sarcopenia composite targets. Table S10. The affinity energy of Honokiol-AKT1. Table S11. The affinity energy of Magnolol-AKT1. Table S12. The affinity energy of Honokiol-EGFR. Table S13. The affinity energy of Magnolol-EGFR. Table S14. The affinity energy of Honokiol-INS. Table S15. The affinity energy of Magnolol-INS. Table S16. The affinity energy of Obovatol-PIK3CA. Figure S1. Sarcopenia related pathogenic gene products involve in EGFR tyrosine kinase inhibitor resistance (hsa01521). Figure S2. Sarcopenia related pathogenic gene products involve in endocrine resistance (hsa01522). Figure S3. Sarcopenia related pathogenic gene products involve in longevity regulating pathway (hsa04211). Figure S4. The GO and KEGG analysis of core sarcopenia-related pathogenic proteins. Figure S5. Core sarcopenia related pathogenic gene products involve in PI3K-Akt signaling pathway (hsa04151). Figure S6. Core sarcopenia related pathogenic gene products involve in longevity regulating pathway (hsa04213). [file 12863_2022_1029_MOESM1_ESM.zip › Supplementary Figure S1.png]

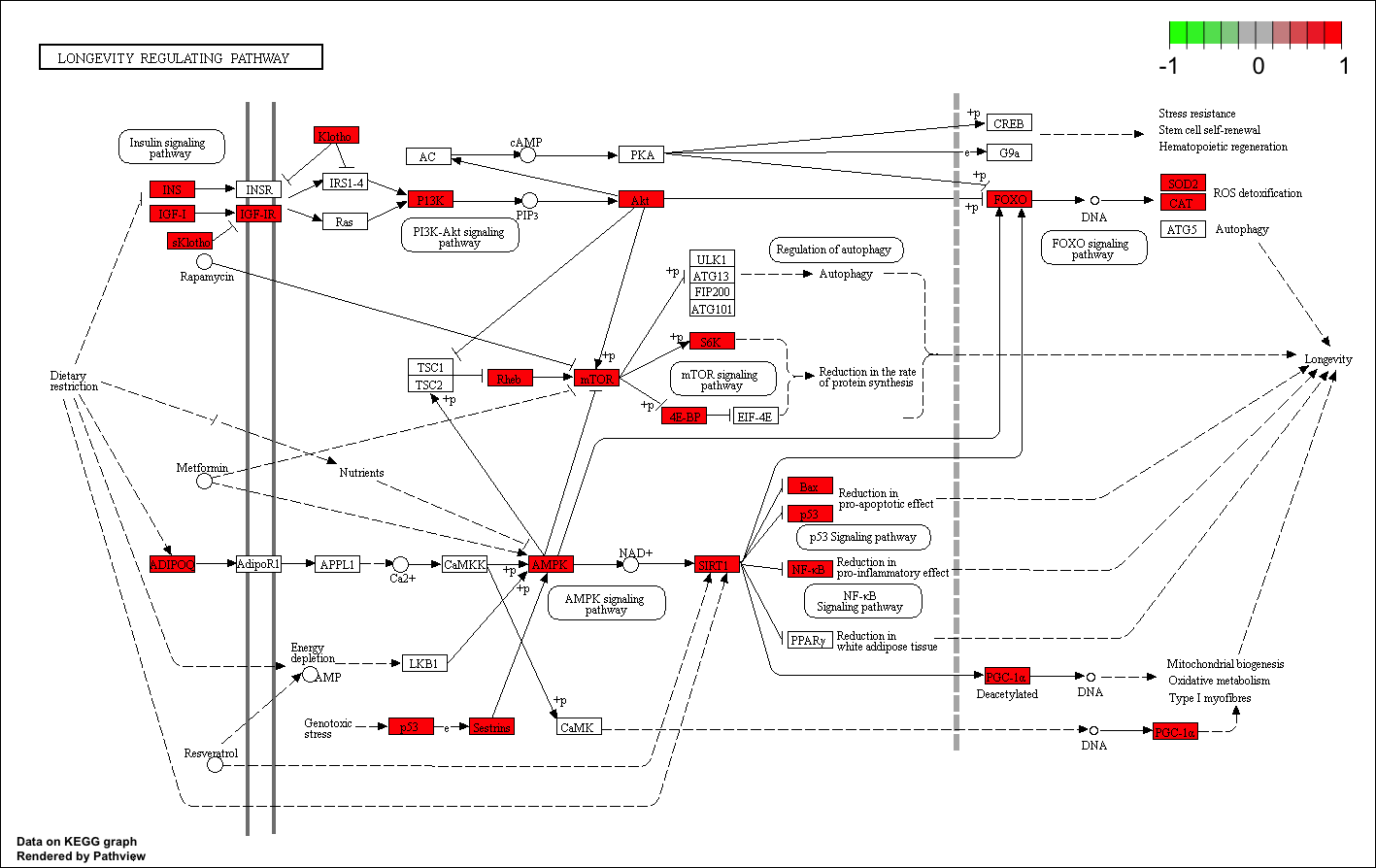

Supplement: Supplementary file 1 — Additional file 1: Table S1. Differentially expressed genes related to sarcopenia in old people of GEO series.Table S2. Integrated data of sarcopenia related pathogenic genes. Table S3. Result of GO enrichment analysis for sarcopenia related pathogenic gene products. Table S4. Result of KEGG enrichment analysis for sarcopenia related pathogenic gene products. Table S5. Result of targets prediction of MC. Table S6. Composite targets of MC and sarcopenia. Table S7. Result of GO enrichment analysis for composite targets of MC and sarcopenia. Table S8. Result of KEGG enrichment analysis for composite targets of MC and sarcopenia. Table S9. Core proteins of MC-sarcopenia composite targets. Table S10. The affinity energy of Honokiol-AKT1. Table S11. The affinity energy of Magnolol-AKT1. Table S12. The affinity energy of Honokiol-EGFR. Table S13. The affinity energy of Magnolol-EGFR. Table S14. The affinity energy of Honokiol-INS. Table S15. The affinity energy of Magnolol-INS. Table S16. The affinity energy of Obovatol-PIK3CA. Figure S1. Sarcopenia related pathogenic gene products involve in EGFR tyrosine kinase inhibitor resistance (hsa01521). Figure S2. Sarcopenia related pathogenic gene products involve in endocrine resistance (hsa01522). Figure S3. Sarcopenia related pathogenic gene products involve in longevity regulating pathway (hsa04211). Figure S4. The GO and KEGG analysis of core sarcopenia-related pathogenic proteins. Figure S5. Core sarcopenia related pathogenic gene products involve in PI3K-Akt signaling pathway (hsa04151). Figure S6. Core sarcopenia related pathogenic gene products involve in longevity regulating pathway (hsa04213). [file 12863_2022_1029_MOESM1_ESM.zip › Supplementary Figure S3.png]

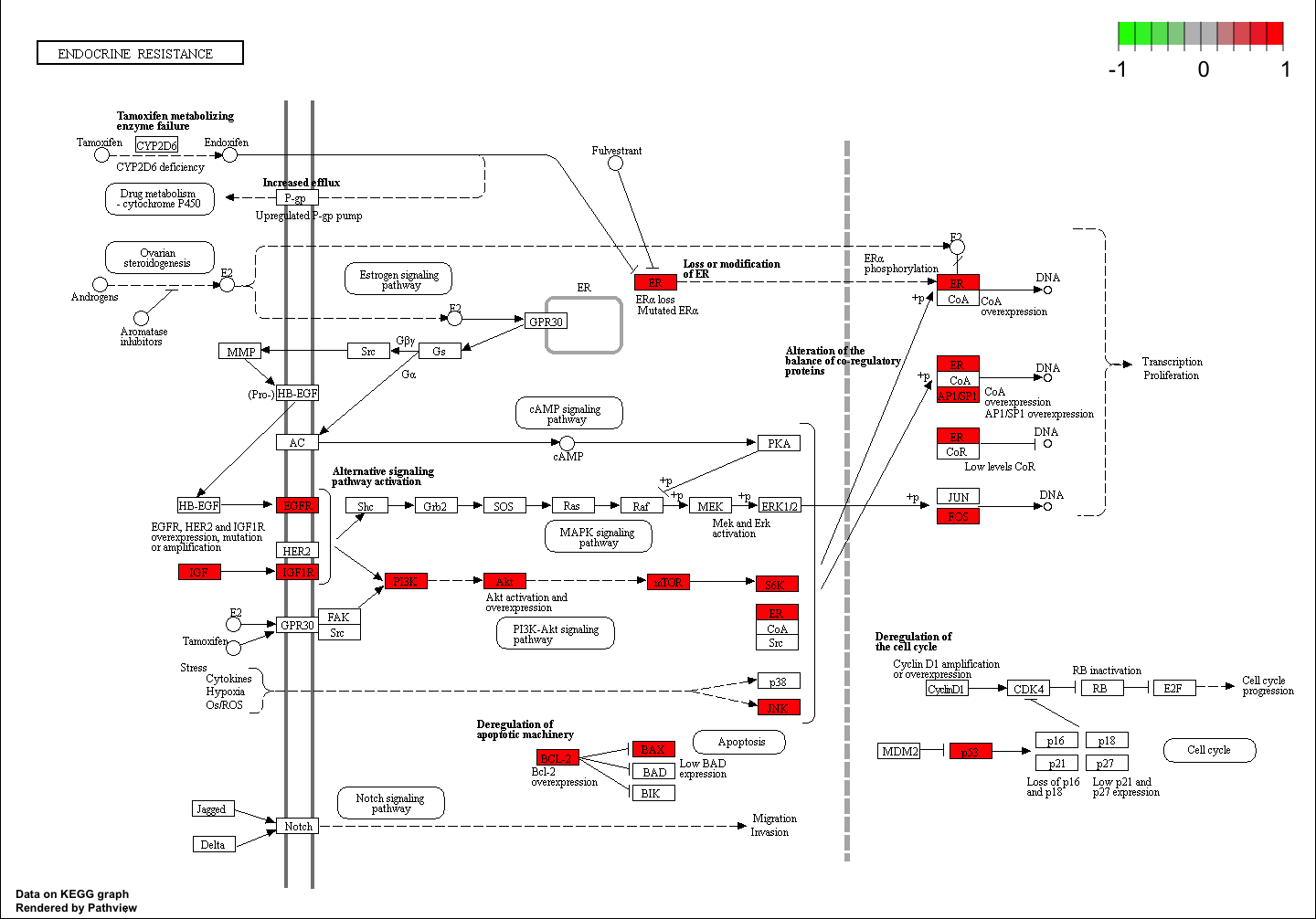

Supplement: Supplementary file 1 — Additional file 1: Table S1. Differentially expressed genes related to sarcopenia in old people of GEO series.Table S2. Integrated data of sarcopenia related pathogenic genes. Table S3. Result of GO enrichment analysis for sarcopenia related pathogenic gene products. Table S4. Result of KEGG enrichment analysis for sarcopenia related pathogenic gene products. Table S5. Result of targets prediction of MC. Table S6. Composite targets of MC and sarcopenia. Table S7. Result of GO enrichment analysis for composite targets of MC and sarcopenia. Table S8. Result of KEGG enrichment analysis for composite targets of MC and sarcopenia. Table S9. Core proteins of MC-sarcopenia composite targets. Table S10. The affinity energy of Honokiol-AKT1. Table S11. The affinity energy of Magnolol-AKT1. Table S12. The affinity energy of Honokiol-EGFR. Table S13. The affinity energy of Magnolol-EGFR. Table S14. The affinity energy of Honokiol-INS. Table S15. The affinity energy of Magnolol-INS. Table S16. The affinity energy of Obovatol-PIK3CA. Figure S1. Sarcopenia related pathogenic gene products involve in EGFR tyrosine kinase inhibitor resistance (hsa01521). Figure S2. Sarcopenia related pathogenic gene products involve in endocrine resistance (hsa01522). Figure S3. Sarcopenia related pathogenic gene products involve in longevity regulating pathway (hsa04211). Figure S4. The GO and KEGG analysis of core sarcopenia-related pathogenic proteins. Figure S5. Core sarcopenia related pathogenic gene products involve in PI3K-Akt signaling pathway (hsa04151). Figure S6. Core sarcopenia related pathogenic gene products involve in longevity regulating pathway (hsa04213). [file 12863_2022_1029_MOESM1_ESM.zip › Supplementary Figure S2.png]
